# Admission Hyperglycemia as an Early Predictor of Severity and Poor Prognosis in COVID-19: A Retrospective Cohort Study of Hospitalized Adults

**DOI:** 10.3390/jcm14207289

**Published:** 2025-10-15

**Authors:** Ligia Rodina, Vlad Monescu, Lavinia Georgeta Caplan, Maria Elena Cocuz, Victoria Bîrluțiu

**Affiliations:** 1 Department of Infectious Diseases, Faculty of Medicine, Lucian Blaga University of Sibiu, 550169 Sibiu, Romania; victoria.birlutiu@ulbsibiu.ro; 2 Clinical Hopsital of Pneumology and Infectious Diseases of Brasov, 500118 Brasov, Romania; laviniacaplandara@yahoo.com (L.G.C.); maria.cocuz@unitbv.ro (M.E.C.); 3 Faculty of Mathematics and Computer Science, Transilvania University of Brasov, 500091 Brasov, Romania; monescu@unitbv.ro; 4 Department of Infectious Disease, Faculty of Medicine, Transilvania University of Brasov, 500036 Brasov, Romania; 5 Department of Clinical Medicine II, County Emergency Clinical Hospital, Lucian Blaga University of Sibiu, 550169 Sibiu, Romania

**Keywords:** COVID-19, SARS-CoV-2, admission hyperglycemia, severity, mortality, ICU

## Abstract

**Background/Objectives**: Admission hyperglycemia is frequent in COVID-19, reflecting stress hyperglycemia, systemic inflammation, and potential viral injury to pancreatic β-cells. It may serve as an early marker of severity. We assessed whether admission hyperglycemia predicts severe disease and poor outcomes in adults without diabetes. **Methods**: We performed a retrospective cohort study including adults hospitalized with RT-PCR/antigen-confirmed COVID-19 between August 2020 and July 2021. Patients < 18 or >80 years, with prior diabetes, or on corticosteroids were excluded. Hyperglycemia was defined as fasting glucose > 106 mg/dL and classified as mild (107–180 mg/dL), moderate (181–300 mg/dL), and severe (>300 mg/dL). Clinical, laboratory, imaging, treatment, utilization, and cost parameters were analyzed. **Results**: Of 1009 patients, 734 (72.7%) were hyperglycemic at admission. Compared with normoglycemic patients, hyperglycemics more often developed respiratory failure (67.7% vs. 38.2%), required CPAP (9.4% vs. 1.5%), and had severe/critical disease (46.9% vs. 25.1%), ICU transfer (6.5% vs. 1.5%), and mortality (3.8% vs. 1.1%) (all *p* ≤ 0.0256). They also showed lymphopenia, eosinopenia, higher inflammatory and coagulation markers, longer hospitalization (12.1 vs. 10.1 days), and increased costs (EUR 1846 vs. 1043) (all *p* < 0.001). Severe hyperglycemia (>300 mg/dL) strongly correlated with inflammation, coagulopathy, tissue injury, and radiologic severity. **Conclusions**: Admission hyperglycemia is a robust, easily measurable predictor of severe COVID-19 and adverse outcomes in non-diabetic adults and is associated with greater resource utilization and higher costs. Early identification may improve risk stratification. Future prospective studies should determine whether early detection and aggressive glycemic control can modify prognosis.

## 1. Introduction

The COVID-19 pandemic caused by SARS-CoV-2 has posed a major global health threat. Since its emergence in late 2019, the virus has resulted in more than 750 million confirmed cases and over 6.9 million reported deaths worldwide, according to the World Health Organization (WHO) [1]. However, excess mortality analyses suggest that the actual number of deaths attributable to COVID-19 is substantially higher, exceeding 14 million globally during 2020–2021 [2]. The burden of disease has varied across continents, with Europe and the Americas experiencing the highest mortality rates, while Africa and parts of Asia have reported lower case fatality ratios, likely influenced by differences in healthcare infrastructure, demographic structure, and underreporting [3,4]. Comparative analyses also show that mortality patterns differ significantly between high-income and low and middle-income countries [5].

The virus continues to spread worldwide, producing heterogeneous clinical phenotypes whose severity is influenced by age, comorbidities, and vaccination status [6,7]. Although the respiratory tract is the primary portal of entry and site of viral replication, leading to diverse pulmonary manifestations, SARS-CoV-2 can also affect the cardiovascular, gastrointestinal, hepatic, pancreatic, renal, thyroid, and central nervous systems [8,9,10,11,12,13].

Patients with COVID-19, particularly older adults and those with comorbidities such as diabetes mellitus (DM), cardiovascular disease, or obesity, are at increased risk of severe disease, intensive care admission, and death [14,15,16,17]. Numerous studies have confirmed that diabetes confers a higher risk of severity and mortality compared with non-diabetic patients [18,19]. Hyperglycemia, the defining feature of DM, has also been associated with adverse outcomes in COVID-19 [19,20,21]. Importantly, several reports indicate that hyperglycemia at hospital admission is frequent even in patients without known diabetes [22,23].

It is increasingly recognized that hyperglycemia arising in the context of acute illness, independent of pre-existing diabetes, reflects more than a transient stress response and serves as a marker of disease severity and an independent predictor of mortality in a variety of infectious and inflammatory conditions, including COVID-19 [10,11,24]. Beyond stress hyperglycemia, SARS-CoV-2 infection appears to cause profound disruption of glucose homeostasis through mechanisms such as exaggerated systemic inflammation with massive cytokine release [25,26,27], direct pancreatic β-cell injury [28,29,30], and stress-related hormonal imbalances [31,32]. Activation of the hypothalamic-pituitary-adrenal axis and increased secretion of cortisol, catecholamines, and glucagon contribute to transient hyperglycemia with negative prognostic impact, even in previously normoglycemic individuals [33,34].

Newly detected hyperglycemia after SARS-CoV-2 infection was one of the notable disturbances observed during the pandemic [9,29,33,34]. However, it remains uncertain whether this represents unrecognized pre-existing dysglycemia, such as prediabetes unmasked by infection [9,12], or de novo diabetes directly caused by COVID-19 [35]. This hyperglycemia may present atypically, sometimes with severe forms, and survivors who experienced hyperglycemia during the acute phase have a significantly increased risk of developing diabetes within one year [29]. Consequently, long-term monitoring is essential for early identification of individuals at risk and for preventing late complications [31,32].

Stress-induced hyperglycemia is a well-described response to severe acute infections and correlates with systemic inflammation [26], endothelial dysfunction [36], thrombosis [36,37], and immune dysregulation [38]. In SARS-CoV-2 infection, available evidence indicates that admission hyperglycemia may serve as an early and easily measurable marker of disease severity, being associated with higher complication rates, intensive care requirements, and mortality, even in patients without known diabetes [39].

**Figure 1:**
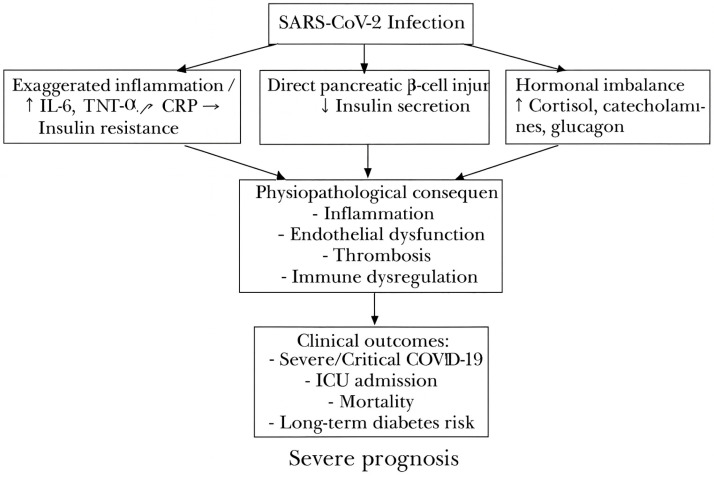
Proposed pathophysiological mechanisms linking SARS-CoV-2 infection to admission hyperglycemia, its systemic consequences (inflammation, endothelial dysfunction, thrombosis, immune dysregulation), and adverse clinical outcomes (severe COVID-19, ICU admission, mortality, long-term diabetes risk). Exaggerated inflammation (↑ IL-6, TNF-α, CRP) → Increased insulin resistance.

The present study aimed to evaluate the prevalence and prognostic value of admission hyperglycemia in adult patients hospitalized with COVID-19 in a tertiary infectious diseases hospital. Specifically, we sought to assess its association with clinical severity, laboratory and imaging parameters, outcomes, resource utilization, and healthcare costs. (Figure 1).

## 2. Materials and Methods

### 2.1. Study Design and Setting

We conducted a retrospective, single-center cohort study at the Clinical Hospital of Pneumophthisiology and Infectious Diseases, Brașov, Romania. The cohort was selected from the institutional database and included 1127 patients hospitalized with COVID-19, confirmed by RT-PCR or antigen testing for SARS-CoV-2, between 1 August 2020 and 31 July 2021. After excluding cases with incomplete or inconsistent data, the final analytical cohort included 1009 patients. The study was approved by the Ethics Committee of the Clinical Hospital of Pneumophthisiology and Infectious Diseases, Brașov (approval no. 9328/20 June 2024) and by the Ethics Committee of the “Lucian Blaga” University of Sibiu (approval no. 16/25 November 2022). COVID-19 diagnosis was based on clinical presentation and confirmed by laboratory testing. All patient data were anonymized prior to analysis. Data extraction from administrative databases and medical records was performed by the first author. Admission fasting glucose was measured using a Konelab 60i automated analyzer (Thermo Scientific, Vantaa, Finland) (based on the enzymatic glucose-hexokinase method with photometric detection. In this method, glucose is phosphorylated by hexokinase and subsequently oxidized by glucose-6-phosphate dehydrogenase (G6PD) in the presence of NAD^+^/NADP^+^. The reaction generates NADH/NADPH, directly proportional to glucose concentration, which is quantified at 340 nm. The analyzer automatically dispenses reagents, performs photometric measurements, and reports results in mg/dL.

### 2.2. Statistical Analysis

All analyses were conducted using MATLAB R2024a (MathWorks, Natick, MA, USA, Statistics Toolbox), Microsoft Office 365 Excel, and Python 3.11 (Python Software Foundation, Beaverton, OR, USA). Python packages included pandas for data wrangling, tabulation, and cohort filtering; numpy for numerical operations; and scipy.stats for inferential statistics.

Descriptive statistics, cross-tabulations, and hypothesis testing were performed within this environment, with plots and tables generated in Python and subsequently reviewed and formatted in Excel. Comparisons of proportions across glycemic groups were performed using chi-square tests of independence (2 × 2), as outcomes of interest (e.g., abnormal vs. normal laboratory values, treatment received vs. not received) were categorical. This approach is appropriate for testing associations between categorical variables when expected counts are adequate.

Within hyperglycemia severity subgroups, relationships between continuous laboratory parameters and binary-encoded clinical variables were explored using Spearman rank correlations. Spearman’s method was selected because it is robust to non-normality and differences in measurement scales, which are frequent in clinical datasets. *p*-values were computed using the scipy.stats functions in Python and verified with MATLAB correlation functions for consistency.

### 2.3. Aim and Objectives

The primary aim of this study was to evaluate admission hyperglycemia as an early predictor of severity and poor prognosis in non-diabetic patients with COVID-19. The specific objectives were to (i) determine the prevalence of admission hyperglycemia and assess its association with demographic, clinical, laboratory, imaging, and outcome parameters; (ii) examine its relationship between the degree of admission hyperglycemia (mild, moderate, severe) and the risk of progression to severe or critical COVID-19, including the need for intensive interventions and mortality; (iii) analyze the correlation of admission hyperglycemia with inflammatory, coagulation, and tissue injury biomarkers; and (iv) evaluate the financial impact of admission hyperglycemia in terms of hospitalization duration, ICU transfer, and healthcare costs.

### 2.4. Inclusion and Exclusion Criteria

Eligible participants were hospitalized adults (>18 years) with confirmed SARS-CoV-2 infection and documented glucose levels at admission. Exclusion criteria were age < 18 or >80 years, pre-existing diagnosis of type 1 or type 2 diabetes mellitus, and corticosteroid therapy prior to admission.

### 2.5. Treatments and Clinical Severity Classification

Standard in-hospital management included oxygen therapy (low- or high-flow), non-invasive ventilation (CPAP), and pharmacological treatments such as lopinavir/ritonavir, favipiravir, remdesivir, hydroxychloroquine, glucocorticoids, tocilizumab, anakinra, and insulin. COVID-19 severity was classified according to national and international protocols [40,41]. Mild cases included patients with upper respiratory tract symptoms without hypoxemia or pneumonia; moderate cases were defined as imaging-confirmed pneumonia without hypoxemia and oxygen saturation (SpO_2_) > 94%. Severe disease was defined as imaging-confirmed pneumonia with at least one of the following: respiratory rate > 30/min, severe respiratory distress, or SpO_2_ < 90%. Critical cases included patients with respiratory failure requiring ventilatory support, acute respiratory distress syndrome (ARDS), sepsis, septic shock, or acute thrombotic events.

### 2.6. Definition of Study Groups and Outcomes

Patients were divided into two groups: those with admission glucose > 106 mg/dL (hyperglycemia) and those with glucose < 106 mg/dL (normoglycemia). Hyperglycemia was further stratified into mild (107–180 mg/dL), moderate (181–300 mg/dL), and severe (>300 mg/dL). Associations with demographic, medical history, clinical, laboratory, imaging, and outcome parameters were analyzed across groups.

### 2.7. Epidemiological and Clinical Variables

A comprehensive set of variables were analyzed and grouped into epidemiological, clinical, laboratory, imaging, and economic domains. Table 1 provides an overview of the variables included and the rationale for their selection.

## 3. Results

### 3.1. Demographic and Prognostic Characteristics

**Figure 2:**
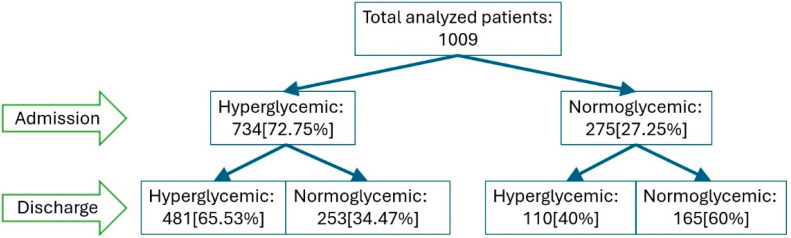
Changes in glycemic status from admission to discharge among patients with COVID19. Legend: Flowchart illustrating the evolution of glycemic status from admission to discharge among 1009 patients with COVID-19.

From the initial cohort of 1127 patients, after excluding incomplete or inconsistent data, 1009 patients were analyzed: 734 (72.7%) were hyperglycemic at admission, while 275 (27.2%) were normoglycemic. During hospitalization, 34.4% of hyperglycemic patients became normoglycemic by discharge, whereas 40% of normoglycemic patients developed hyperglycemia (Figure 2).

**Figure 3:**
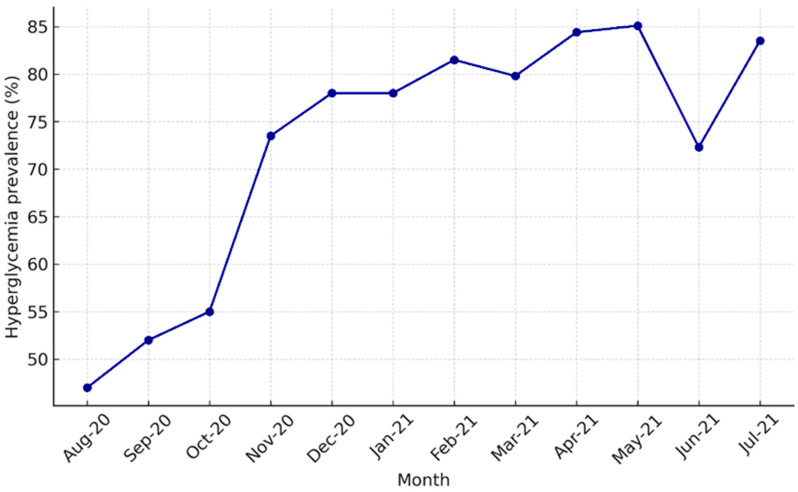
Monthly prevalence of admission hyperglycemia among hospitalized COVID-19 patients. Legend: Monthly prevalence of admission hyperglycemia among hospitalized COVID-19 patients between August 2020 and July 2021. A marked increase was observed in November 2020 (second pandemic wave) and between February–May 2021 (Alpha wave).

Temporal analysis (August 2020–July 2021) showed a steady increase in admission hyperglycemia, with a peak between February and May 2021. The November 2020 rise coincided with the beginning of the second pandemic wave, characterized by more severe cases, and the February–May 2021 peak corresponded to the Alpha wave, also marked by more severe disease. This suggests a possible association between hyperglycemia and severe COVID-19 (Figure 3).

Sex distribution differed significantly: men predominated in the hyperglycemic group (58.3%), whereas women predominated in the normoglycemic group (54.5%, *p* = 0.0003). Hypertension was significantly more frequent in hyperglycemic than in normoglycemic patients (51.2% vs. 41.5%). Other cardiovascular conditions (myocardial infarction, coronary stent) were less common, without significant differences. COPD prevalence was similar between groups; asthma was slightly more frequent in normoglycemics, without statistical significance. Obesity was significantly more common in hyperglycemics (10.8% vs. 3.6%), underscoring the link between body weight status and dysglycemia at admission. Hepatic steatosis was infrequent, with no notable group differences (Table 2).

Age distribution showed clear differences: younger patients (36–50 years) were more often normoglycemic, whereas older patients (66–80 years) were predominantly hyperglycemic, with statistically significant results (Table 3).

Regarding symptoms, the most common (in descending order) were fever, cough, dyspnea, fatigue, and myalgia. Cough was significantly more prevalent in hyperglycemics, and dyspnea was far more frequent in the same group (47.3% vs. 27.6%), with highly significant differences. Headache, pharyngitis, and taste/smell disturbances were more common in normoglycemics, without statistical significance. Gastrointestinal symptoms (nausea, vomiting, diarrhea) were significantly more frequent in normoglycemics (Table 4).

Age-stratified CPAP need showed that patients < 35 years did not require ventilatory support regardless of glycemic status. In the 51–65 and 66–80 age groups, CPAP need was significantly higher in hyperglycemics (Table 5).

Overall, hyperglycemics developed acute respiratory failure more often (67.7% vs. 38.2%), required CPAP more frequently (9.4% vs. 1.5%), and had higher rates of ICU transfer (6.5% vs. 1.5%) and mortality (3.8% vs. 1.1%). Disease severity also varied significantly: mild cases were more frequent in normoglycemics (40.4%), while severe and critical forms predominated in hyperglycemics (40.2% and 6.8%, respectively) (Table 6).

Length of stay was significantly longer in hyperglycemics (12.1 vs. 10.1 days), and mean hospitalization costs were substantially higher (€1846 vs. €1043) (Table 7).

### 3.2. Laboratory Findings

Laboratory parameters differed significantly. Leukopenia and thrombocytopenia had similar frequencies. Lymphopenia (<1000/μL) was much more frequent in hyperglycemics (59.6% vs. 23.1%, *p* < 0.001), as was eosinopenia (84.2% vs. 41.7%, *p* < 0.001). Inflammatory and severity markers were significantly more often elevated in hyperglycemics: ESR > 10 mm/h (82.1% vs. 67.2%, *p* < 0.001), CRP > 10 mg/L (76.6% vs. 60.6%, *p* < 0.001), ferritin > 250 ng/mL (81.7% vs. 56.4%, *p* < 0.001), and LDH > 245 U/L (89.4% vs. 78.1%, *p* < 0.001). Coagulation/inflammation parameters were more often abnormal: D-dimer > 243 ng/mL (62.6% vs. 49.2%, *p* < 0.001) and fibrinogen > 450 mg/dL (59.0% vs. 45.3%, *p* < 0.001). ALT > 45 U/L (48.6% vs. 30.3%, *p* < 0.001) and AST > 45 U/L (38.5% vs. 25.5%, *p* < 0.001) were also more frequent with hyperglycemia. Creatinine did not differ significantly (Table 8)

### 3.3. Treatments

Pharmacological management included the use of antivirals, immunomodulatory agents, and corticosteroids, with relevant differences between groups.

Antiviral therapy varied significantly: remdesivir and favipiravir were more frequently administered to hyperglycemic patients, whereas lopinavir/ritonavir (Kaletra) was prescribed more often in the normoglycemic group. Hydroxychloroquine use showed no notable differences (Table 9).

Immunomodulatory therapy targeting cytokine storm control (tocilizumab and anakinra) was also significantly more common in hyperglycemic patients, suggesting a higher burden of systemic inflammation and more severe disease progression in this subgroup (Table 10).

Corticosteroid therapy was strongly associated with the development of in-hospital hyperglycemia. Among the 275 patients who were normoglycemic at admission, 40% developed hyperglycemia during hospitalization, and 94.5% of these had received corticosteroids. Although new-onset hyperglycemia was common, it was not consistently associated with worse outcomes: only 16.2% of these patients progressed to severe or critical forms, and just 0.9% required non-invasive ventilation or ICU transfer.

Nevertheless, persistent dysglycemia was observed at discharge, with 29.3% of patients presenting glucose levels > 140 mg/dL, highlighting potential long-term metabolic consequences and the importance of follow-up and preventive interventions (Table 11).

### 3.4. Relationship Between Degree of Admission Hyperglycemia and Risk of Progression to Severe COVID-19

Among hyperglycemics (*n* = 734), 571 (77.7%) had 107–180 mg/dL, 141 (19.2%) had 181–300 mg/dL, and 22 (2.9%) had > 300 mg/dL at admission. To better understand the impact of hyperglycemia on clinical and inflammatory profiles, Spearman correlations among biological and clinical parameters were analyzed across the three glycemic strata. Severe hyperglycemia (≥300 mg/dL) was associated with an accentuated systemic inflammatory response and more severe pulmonary, vascular, and hematologic involvement compared with moderate or mild hyperglycemia (Table 12).

**Figure 4:**
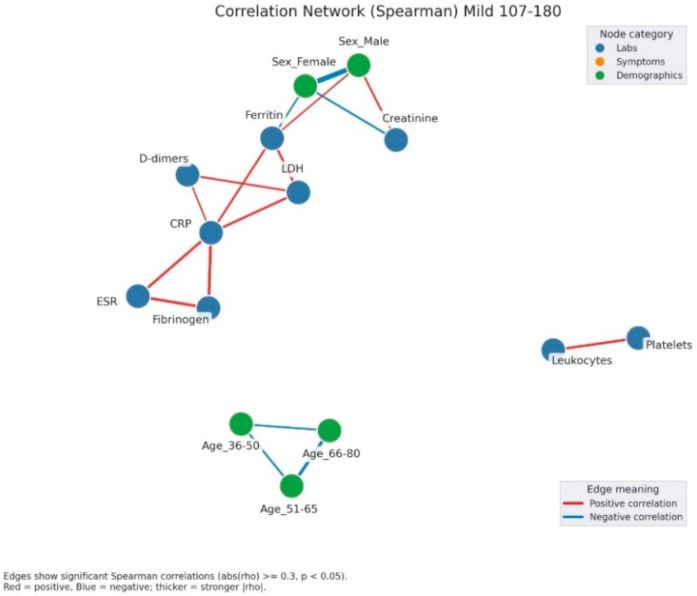
Correlation network of clinical, laboratory, and demographic parameters in patients with mild admission hyperglycemia (107–180 mg/dL). Legend: The network illustrates significant correlations (|ρ| ≥ 0.3, *p* < 0.05) between laboratory parameters (blue nodes) and demographic factors (green nodes). Red edges = positive correlations, blue edges = negative correlations. Edge thickness reflects correlation strength. A well-defined inflammatory cluster can be observed, linking CRP, fibrinogen, D-dimers, LDH, ferritin, and ESR, with strong positive interconnections among these markers. Male sex shows a negative correlation with ferritin and a positive one with creatinine, suggesting sex-related differences in the biological response to infection. In the lower part, age-related variables form a small group of negative correlations, reflecting the distinct distribution of age subgroups.

Correlation network analysis demonstrated a progressive increase in structural complexity from mild to severe hyperglycemia. In mild hyperglycemia (Figure 4), the network remained relatively simple, with correlations primarily restricted to inflammatory markers (CRP, ESR, fibrinogen, ferritin, LDH) and hematological parameters (leukocyte and lymphocyte counts). Clinical symptoms appeared scattered and weakly connected.

**Figure 5:**
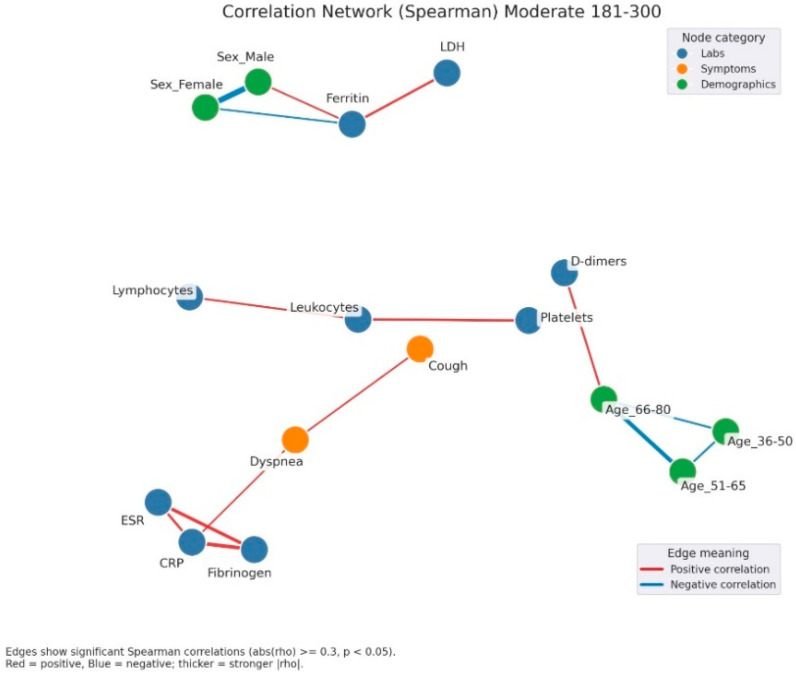
Correlation network of clinical, laboratory, and demographic parameters in patients with moderate admission hyperglycemia (181–300 mg/dL). Legend: Correlation network in patients with moderate hyperglycemia (181–300 mg/dL). Nodes represent laboratory parameters (blue), symptoms (orange), and demographic factors (green). Edges indicate significant Spearman correlations (|rho| ≥ 0.3, *p* < 0.05). Red edges = positive correlations; blue edges = negative correlations. Edge thickness reflects correlation strength. The network here shows a stronger integration between inflammation and clinical manifestations. A dense cluster connects CRP, fibrinogen, and ESR, all positively correlated, and extending toward symptoms such as dyspnea and cough, suggesting the link between systemic inflammation and respiratory impairment. Leukocytes and lymphocytes are interconnected, with further links to cough and platelet count, reflecting immune activation. Ferritin shows positive correlations with LDH and male sex, again pointing to sex-related variations in inflammatory response.

In moderate hyperglycemia (Figure 5), the inflammatory-hematological cluster became more consolidated and expanded through the integration of coagulation markers (D-dimer) and renal function parameters (creatinine), particularly among older patients. Clinical symptoms remained heterogeneous, with limited interconnections.

**Figure 6:**
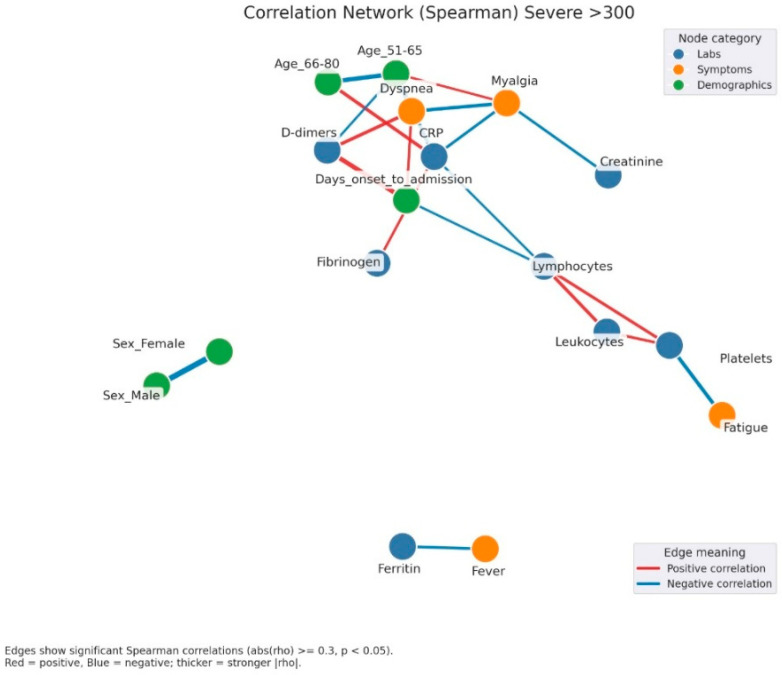
Correlation network of clinical, laboratory, and demographic parameters in patients with severe admission hyperglycemia (>300 mg/dL). Legend: Correlation network in patients with severe hyperglycemia (glucose > 300 mg/dL). Nodes represent clinical variables, categorized as laboratory parameters (blue), symptoms (orange), or demographic factors (green). Edges indicate significant Spearman correlations (|rho| ≥ 0.3, *p* < 0.05). Red edges = positive correlations; blue edges = negative correlations. Thicker edges = stronger correlation strength. A complex and highly interconnected cluster is evident, linking CRP, fibrinogen, D-dimers, and dyspnea, which also correlate with myalgia and the duration from symptom onset to hospital admission. This pattern suggests that the inflammatory burden and delayed presentation are key contributors to disease severity. Lymphocyte and leukocyte counts show opposite trends, with negative correlations to inflammatory markers, reflecting immune exhaustion and dysregulation typical of severe infection. Additionally, fatigue and platelet count form another small cluster, possibly associated with endothelial dysfunction and systemic inflammation. The separation of ferritin and fever into an isolated subnetwork may reflect late-phase inflammatory activity.

In severe hyperglycemia (Figure 6), the correlational structure was markedly dense, comprising numerous positive and negative associations. Inflammatory, hematological, and coagulation markers formed a central cluster interconnected with demographic variables and clinical manifestations such as fever, dyspnea, and asthenia. Negative correlations, including those between lymphocytes and platelets, were more frequent, suggesting profound immunological and hematological imbalance.

**Figure 7:**
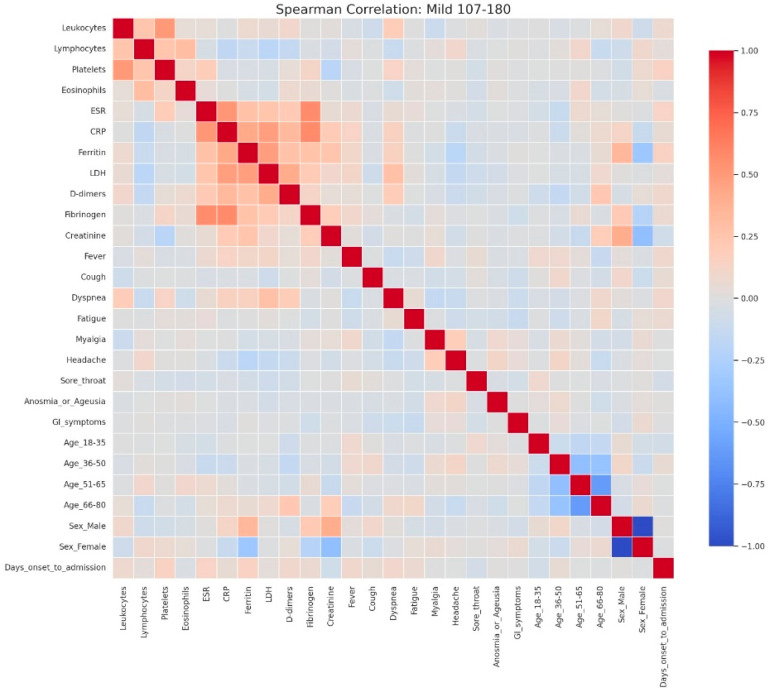
Spearman correlation heatmap of clinical, laboratory, and demographic parameters in patients with mild admission hyperglycemia (107–180 mg/dL). Legend: Clinical, demographic, and laboratory variables are displayed along both axes. Color intensity reflects correlation strength and direction (red = positive, blue = negative, neutral associations shown in light gray). The heatmap shows moderate positive correlations among inflammatory markers, including CRP, ESR, fibrinogen, LDH, ferritin, and D-dimers, reflecting the low-grade but coordinated inflammatory response even in mild disease. A weak positive relationship between leukocyte count and platelets is also observed, consistent with mild systemic activation.Demographic variables show limited correlation with laboratory parameters, though male sex is weakly associated with higher ferritin levels, while female sex and younger age groups show slight inverse correlations. The age subgroups display mutual negative correlations, as expected due to categorical separation.No strong links are seen between inflammatory markers and clinical symptoms in mild cases, suggesting that symptom burden (e.g., cough, fatigue, sore throat) may not directly mirror measurable systemic inflammation at this disease stage.

**Figure 8:**
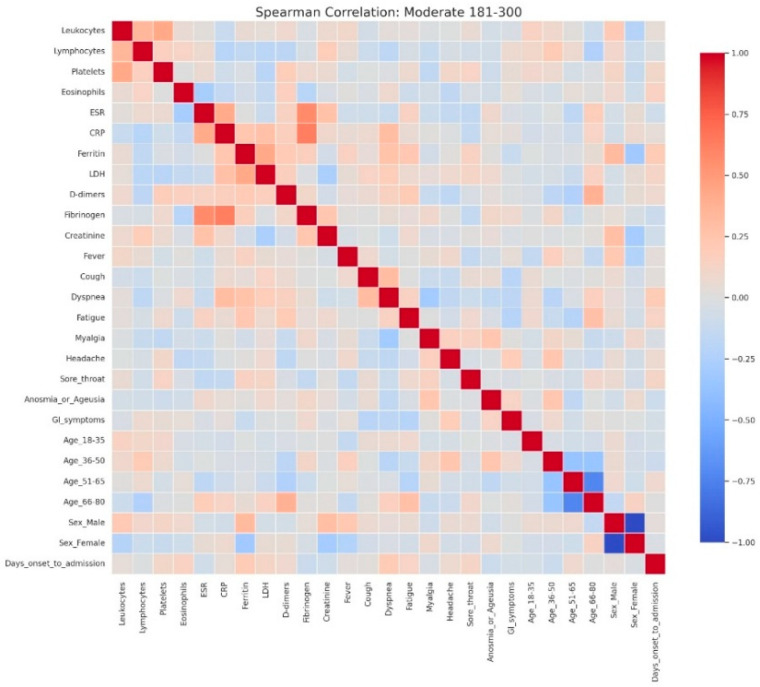
Spearman correlation heatmap of clinical, laboratory, and demographic parameters in patients with moderate admission hyperglycemia (181–300 mg/dL). Legend: Matrix of correlations across variables. Positive correlations are shown in red and negative correlations in blue, with darker colors indicating stronger associations, and neutral associations in light gray. The moderate cohort exhibits a clearer clustering of inflammatory markers, including CRP, ESR, fibrinogen, D-dimers, LDH, and ferritin, showing moderate-to-strong positive correlations—consistent with a heightened systemic inflammatory response. Weak inverse associations appear between inflammatory parameters and lymphocyte counts, reflecting early immune suppression.Slight positive correlations between CRP and symptoms such as dyspnea and cough indicate that clinical manifestations begin to parallel biochemical inflammation as disease severity increases. Demographic variables such as male sex and older age (51–80 years) show mild positive correlations with inflammatory markers, consistent with known risk profiles for more severe COVID-19.

**Figure 9:**
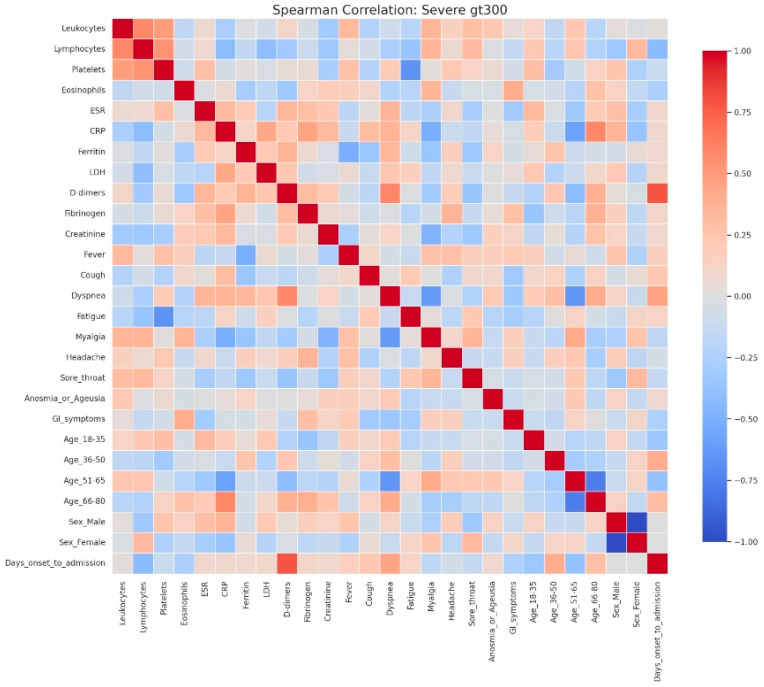
Spearman correlation heatmap of clinical, laboratory, and demographic parameters in patients with severe admission hyperglycemia (>300 mg/dL). Legend: Comprehensive visualization of correlations between clinical, demographic, and laboratory parameters. Strong positive correlations appear in dark red, while strong negative correlations appear in dark blue. In this group, inflammatory and biochemical markers (CRP, ESR, ferritin, LDH, D-dimers, and fibrinogen) show intense positive correlations, reflecting the pronounced systemic inflammatory response characteristic of severe disease. These markers also exhibit negative correlations with lymphocyte counts, consistent with immune exhaustion and lymphopenia observed in critical COVID-19 cases. Correlations extend beyond laboratory variables: dyspnea, fatigue, and myalgia display moderate positive associations with inflammation-related markers, suggesting that symptom severity parallels biochemical hyperinflammation. Additionally, older age groups (≥51 years) and male sex show weak-to-moderate positive correlations with inflammatory and coagulation parameters, supporting their role as demographic risk factors for severe outcomes. Overall, this heatmap demonstrates a densely interconnected inflammatory network in severe COVID-19, where immune dysregulation, hyperinflammation, and demographic vulnerability converge to amplify disease severity and clinical burden.

Heatmap analyses corroborated this progression, showing moderate associations with a clearly delineated inflammatory cluster in mild hyperglycemia (Figure 7), stronger integration of inflammatory and hematological parameters in moderate hyperglycemia (Figure 8), and an intricate pattern in severe hyperglycemia (Figure 9), characterized by inverse relationships and broader involvement of clinical symptoms.

## 4. Discussion

In this retrospective study of 1.009 adult inpatients with COVID-19, admission hyperglycemia was associated with significantly more severe clinical courses, higher medical resource consumption, and worse prognosis than normoglycemia. Our results align with the literature identifying admission hyperglycemia as an important predictor of severity and mortality in COVID-19 [6,7,8,19]. Admission glucose correlates directly with risk of progression to severe disease and death [39,51]. In line with the study by Sheng-Ping Liu et al., which demonstrated that hyperglycemia is a strong predictor of poor prognosis in COVID-19 [19], our findings confirm the negative prognostic role of admission hyperglycemia. However, our work adds several novel aspects: it is, to our knowledge, the first large-scale study from Eastern Europe analyzing over one thousand hospitalized COVID-19 patients; it stratifies hyperglycemia by severity at admission, allowing a more granular understanding of its clinical implications; and it uniquely assesses healthcare resource utilization and direct hospitalization costs in relation to admission glycemia. These additional insights extend the current knowledge beyond the prognostic associations described by Liu et al. The risk factors identified here are consistent with prior research, highlighting robust correlations between systemic immune responses and inflammation with potential multiorgan involvement that contributes to worsening disease [6,7,8].

### 4.1. Metabolic Profile and Risk Factors

Sex distribution differed significantly by admission glycemia, with men predominating among hyperglycemics (58.3% vs. 41.7%; *p* = 0.0003) and women among normoglycemics (54.5% vs. 45.5%). This matches epidemiologic data indicating higher prevalence and worse outcomes in men with SARS-CoV-2 infection [52]. Age was also significantly associated with admission glycemia: patients aged 36–50 years were more often normoglycemic (24.7% vs. 17.6%), whereas those aged 66–80 years were predominantly hyperglycemic (37.9% vs. 26.5%; *p* < 0.001), in line with evidence that advanced age is a risk factor for hyperglycemia and poor prognosis in SARS-CoV-2 infection [53,54]. CDC guidance likewise emphasizes substantially higher risks of severe disease, complications, and mortality in those ≥65 years [44]. Early glycemic monitoring and metabolic control strategies are therefore crucial in older patients.

Hypertension and obesity were significantly more frequent among hyperglycemics (51.2% vs. 41.5%, *p* = 0.007; and 10.8% vs. 3.6%, *p* = 0.0006, respectively). These comorbidities are well -known for worsening the course of COVID-19, being associated with higher mortality and severe complications [42]. Respiratory comorbidities did not differ significantly, suggesting metabolic comorbidities (hypertension and obesity) are most relevant for hyperglycemic COVID-19 patients [38,42,43,44]. Mechanistically, hyperglycemia impairs immune responses and increases susceptibility to severe infections; obesity, hypertension, and diabetes contribute to severe disease through factors such as increased ACE2 expression in adipose tissue and exacerbation of systemic inflammation [43,51,55].

Similarly, hypertension and cardiovascular disease predispose to endothelial dysfunction and coagulation abnormalities, thereby potentiating the prothrombotic effects of hyperglycemia [42].

Pulmonary diseases, such as COPD and asthma, although less frequent in our cohort, have been described as risk factors for adverse outcomes due to pre-existing airway inflammation and reduced respiratory reserve [38]. Neoplastic disease and its therapies (chemotherapy, corticosteroids, or targeted immunotherapy) may further exacerbate immune suppression and glucose dysregulation. Hepatic steatosis, although infrequent, could contribute to altered glucose and lipid metabolism, amplifying systemic inflammation and insulin resistance.

### 4.2. Clinical Severity and Respiratory Involvement

In our non-diabetic cohort (*n* = 1009), a remarkable 72.7% presented with disordered glucose metabolism at admission, with values from 107 mg/dL (106 mg/dL being the analyzer’s upper normal limit) up to 620 mg/dL (the latter in a 50-year-old woman).

A temporal analysis of COVID-19 hospitalizations and admission hyperglycemia revealed that, in the first months (August-October 2020), hyperglycemia was present in 47–55% of patients, a lower percentage compared to subsequent months. The sharp increase observed between November 2020 and January 2021 (73–78%) coincided with the second pandemic wave and a growing number of severe cases, suggesting that hyperglycemia may represent a marker of COVID-19 severity. The peak values recorded between February and May 2021 (82–85%) corresponded to the wave driven by the Alpha variant and indicated that more than four out of five hospitalized patients presented with admission hyperglycemia, supporting the hypothesis of a bidirectional relationship between SARS-CoV-2 and hyperglycemia: the viral variant associated with more severe forms induces hyperglycemia, while metabolic disturbances, in turn, influence the clinical phenotype of COVID-19. The 38-percentage-point difference between the minimum and maximum values is statistically significant and highlights the clinical relevance of admission hyperglycemia as a prognostic indicator. These findings suggest that admission hyperglycemia is closely associated with COVID-19 severity; thus, early monitoring and glycemic control may be essential to improve outcomes in hospitalized patients [9,10,11,39].

Likewise, in our cohort, severe forms of COVID-19 were more frequent in hyperglycemic patients, and acute respiratory failure was nearly twice as common compared to normoglycemic patients, suggesting more pronounced pulmonary involvement associated with hyperglycemia. Although the overall incidence of critical forms was low, these were more than five times more frequent in hyperglycemics. The need for non-invasive ventilation was nearly five times higher among hyperglycemics, whereas mild forms predominated in normoglycemics. The probability of ICU admission was more than five times higher in hyperglycemic patients, noting that some patients used CPAP in the infectious diseases ward due to ICU bed shortages. Mortality was also significantly higher among hyperglycemics. These data show that admission hyperglycemia is not only a marker of severity but also a possible independent negative prognostic factor, contributing to clinical decompensation and increased risk of death [10,34,35]. The associations persist even in the absence of a previous diagnosis of diabetes, suggesting that stress hyperglycemia or newly diagnosed diabetes may have similar clinical implications [31,39]. In 60.4% of hyperglycemic patients, hyperglycemia persisted during hospitalization; 12.4% required insulin therapy for glycemic control. Among patients with admission glucose > 180 mg/dL, 77.5% developed severe or critical forms with respiratory failure, 16.5% required non-invasive ventilation, and 7% died. In a retrospective study (Inner-City Hospital, 2020), patients without diabetes but with admission glucose > 200 mg/dL had significantly higher mortality; moreover, stress hyperglycemia in the absence of pre-existing diabetes was associated with even greater risks than in patients with known diabetes [56]. Consequently, admission glucose levels correlate directly with clinical severity and risk of death, as reported both in our study and in other research [21,22,51].

Similar results were reported by Fadini et al. in a retrospective analysis of 413 COVID-19 patients, which highlighted a strong correlation between admission glucose and clinical severity/complications, with a significantly stronger association (*p* < 0.001) in newly diagnosed diabetes (HbA1c ≥ 6.5% or random glucose ≥ 11.1 mmol/L [≥200 mg/dL] in the presence of hyperglycemia symptoms) compared to pre-existing diabetes. Each 2 mmol/L (36 mg/dL) increase in fasting admission glucose was associated with a 21% relative increase in the risk of severe disease [21]. Similarly, Coppelli et al., in a retrospective study of 271 patients, showed that admission hyperglycemia remained the only independent predictor of mortality (*p* = 0.04); mortality was significantly higher in patients with “new” hyperglycemia (≥140 mg/dL) without diabetes compared to normoglycemics (<140 mg/dL): 39.4% vs. 16.8% [22].

Age-stratified analysis of CPAP requirement adds further nuance: in patients ≤ 50 years, the need was low and similar between groups, likely reflecting better respiratory reserve; in those aged 51–65 years, hyperglycemia was associated with an eightfold higher risk of requiring CPAP, while in patients ≥ 66 years, the rate was about 4.5 times higher than in normoglycemics, suggesting a possible additive effect of hyperglycemia on age-related pulmonary vulnerability [45].

### 4.3. Immune Dysfunction and Inflammatory Profile in Hyperglycemia

Accumulating evidence indicates that hyperglycemia is associated with profound immune dysfunction, including lymphopenia, elevated CRP and D-dimer, and coagulation abnormalities [57,58]. Recent meta-analyses confirm significantly higher levels of ferritin, CRP, IL-6, fibrinogen, and D-dimer in hyperglycemic/diabetic patients compared with normoglycemics, reflecting an amplified proinflammatory and prothrombotic state [36,59].

In our cohort, laboratory findings consistently linked hyperglycemia with heightened immune/inflammatory responses. Severe lymphopenia (<1000/μL) occurred in nearly 60% of hyperglycemics versus 23% of normoglycemics, underscoring marked cellular immune impairment. Hyperglycemia may exacerbate this dysfunction through mechanisms such as abnormal protein glycosylation and impaired T-cell activation, thereby limiting viral clearance. Supporting these observations, a recent study in *Diseases* (2024) reported that hyperglycemic COVID-19 patients exhibited reduced lymphocytes, lower oxygen saturation, and increased LDH and ferritin, all markers of severe disease [57]. Other reports similarly noted that hyperglycemia and diabetes are associated with profound lymphopenia and elevated CRP, IL-6, TNF-α, and PCT compared with non-diabetic patients [59]. Complete eosinopenia, observed in 84% of hyperglycemics in our study, further supports an intense acute inflammatory response; its predictive value for ICU admission and respiratory support has been described elsewhere [60]. Consistent with these hematologic abnormalities, hyperglycemic patients showed significantly higher levels of ESR, CRP, ferritin, and fibrinogen, suggesting a pronounced proinflammatory milieu driven by oxidative stress, endothelial dysfunction, and activation of the IL-6/TNF-α cytokine axis [57,61]. Elevated D-dimer and LDH indicated increased thrombotic risk and extensive tissue injury, particularly pulmonary, in line with previous reports identifying hyperglycemia as an independent risk factor for thromboembolic complications and alveolo-capillary damage [57,61].

Transaminase elevations (ALT, AST) were also more frequent in hyperglycemics, possibly reflecting systemic inflammatory injury, viral-induced hepatic involvement, metabolic toxicity, or muscle damage [57,58]. In contrast, creatinine did not differ significantly between groups, suggesting that renal impairment at admission is more likely attributable to factors such as hypotension, hypoxia, or nephrotoxic drugs rather than hyperglycemia itself [62].

Taken together, these findings indicate that hyperglycemic patients display a markedly more severe inflammatory and hematologic profile than normoglycemics, characterized by lymphopenia, eosinopenia, and elevated inflammatory and coagulation markers, thereby supporting the concept of hyperglycemia as both a marker and amplifier of severe COVID-19.

### 4.4. Comparative Analysis by Hyperglycemia Severity

The comparative analysis of clinical and biological profiles according to hyperglycemia severity highlights a graded progression of the inflammatory response and hematologic parameters as glucose levels rise. Three distinct subgroups—mild hyperglycemia (107–180 mg/dL), moderate hyperglycemia (181–300 mg/dL), and severe hyperglycemia (>300 mg/dL)—showed significant quantitative and qualitative differences, with important clinical and prognostic implications.

Even in the absence of a prior diabetes diagnosis, elevated glucose levels can amplify systemic inflammation and alter hematologic and coagulation functions [63]. In the severe hyperglycemia group (>300 mg/dL), notable correlations were observed: CRP was inversely associated with oxygen saturation and positively with radiologic severity and D-dimer levels, suggesting an intense systemic inflammatory syndrome with pulmonary and vascular involvement. D-dimers correlated with time to hospitalization and radiologic score, indicating an early, aggressive vascular inflammatory process associated with rapid disease progression. Markers of cellular injury, particularly LDH, showed negative correlations with O_2_ saturation and positive associations with dyspnea in severe hyperglycemia, suggesting extensive pulmonary damage [24].

Across all groups, LDH correlated positively with ferritin, AST, and CRP, but these correlations were strongest in the moderate hyperglycemia subgroup, pointing to multisystem cellular injury. A retrospective study by Kumar et al. (2025) [48] demonstrated that CRP, D-dimer, and IL-6 are independent risk factors for COVID-19 severity, while CRP, D-dimer, LDH, ferritin, and the neutrophil-to-lymphocyte ratio (NLR) are independent predictors of mortality. D-dimer emerged as the most sensitive and specific marker of severity, while LDH was the most reliable predictor of mortality [37,38,39,40,41,42,43,44,45,46,47,48,49,50,51,52,53,54,55,56,57,58,59,60,61,62,63,64,65]. Hepatic involvement was evident in moderate hyperglycemia, reflected by correlations between transaminases and inflammatory markers; however, in extreme hyperglycemia, these associations disappeared, possibly indicating less pronounced secondary hepatic injury or a clinical picture dominated by pulmonary and systemic involvement. Diaz-Louzao C. et al. (2022) examined the temporal relationships between inflammatory markers (CRP, IL-6, D-dimer, lymphocyte count) and hepatic injury markers (AST, ALT, GGT), showing different patterns depending on disease evolution and prognosis [66]. Notably, deceased patients had elevated hepatocellular markers positively correlated with inflammatory markers, whereas in survivors, these correlations became inverse after one week of hospitalization [31,67].

Oxygen saturation decreased in parallel with negative correlations to CRP, LDH, and chest radiologic score across all subgroups, with maximum intensity in patients with glucose ≥ 300 mg/dL, reflecting severe hypoxemia and extensive pulmonary involvement. In this group, the radiologic score was the most interconnected marker, correlating with CRP, ferritin, and D-dimer, supporting the suspicion of severe viral pneumonia and intense inflammation [68].

Correlations between inflammatory markers (CRP-LDH, ferritin-LDH) were evident in groups with lower glucose levels but disappeared in extreme hyperglycemia, possibly reflecting a “saturated” inflammatory response in which traditional biological relationships become attenuated due to profound systemic dysfunction.

These results support the hypothesis that elevated glucose levels, even without pre-existing diabetes, can amplify systemic inflammatory responses and induce hematologic and coagulation changes [48,69]. Patients with glucose > 300 mg/dL showed tighter integration of clinical symptoms with biological parameters, which may explain their higher risk of complications and the need for more aggressive monitoring and intervention.

The persistence of isolated symptoms (anosmia, fatigue), even at high glucose levels, suggests that not all clinical manifestations are directly influenced by metabolic status. In contrast, inflammatory and hematologic parameters appear to be more sensitive indicators of hyperglycemia severity and may serve as prognostic markers.

These observations provide an integrated perspective on the interaction between glucose metabolism and the inflammatory response, supporting a stratified management approach in patients with acute hyperglycemia. In practice, early identification of patients with complex networks of correlations (inflammatory, hematologic, and clinical) may guide timely therapeutic interventions and prevent progression to severe forms or major metabolic decompensation.

Overall, the data support the concept that admission hyperglycemia in COVID-19 is more than a simple metabolic marker, representing an indicator of disease severity. Multiple correlations between glucose, inflammation, pulmonary involvement, and tissue injury suggest that early glycemic control may have significant prognostic implications. These findings align with other studies demonstrating the clear association between hyperglycemia and mortality in COVID-19, even in patients without pre-existing diabetes [17,19,26,62].

In the present study, correlation network analysis revealed that hyperglycemia severity is associated with a progressive increase in the complexity of interactions among biological, clinical, and demographic variables. In mild hyperglycemia, the profile was dominated by correlations between inflammatory markers and hematologic parameters, suggesting that even slightly elevated glucose levels can trigger a detectable inflammatory response. As hyperglycemia advanced to moderate levels, the inflammatory-hematologic cluster became more consolidated, with additional connections to coagulation markers and renal function, particularly in older patients, indicating broader systemic activation and increased vulnerability to metabolic and endothelial dysfunction. In severe hyperglycemia, the correlation structure became dense, with numerous positive and negative associations, reflecting greater immunologic and hematologic dysregulation. The integration of respiratory and general symptoms (fever, dyspnea, fatigue) into the biological core of the network reflects a more severe clinical expression and a closer interplay between inflammatory responses and clinical manifestations. These patterns suggest that hyperglycemia is not merely a marker of severity but actively contributes to remodeling the inflammatory and hematologic response, with direct implications for the clinical phenotype and patient prognosis. Ceriello A. (2020), in “Hyperglycemia and COVID-19: what was known and what is really new?”, supports the hypothesis that hyperglycemia is not just a marker but an active factor exacerbating inflammation and the procoagulant state [32].

### 4.5. Pathophysiological Considerations

The relationship between hyperglycemia and poor prognosis in COVID-19 is complex and bidirectional. SARS-CoV-2 infection can induce hyperglycemia through increased release of counter-regulatory hormones, severe systemic inflammation, and possibly by direct injury to pancreatic β-cells. In turn, hyperglycemia worsens immune dysfunction, increases oxidative stress, promotes endothelial damage, and induces a prothrombotic state, thereby amplifying the pathogenic cascades involved in severe disease [10,11,25].

A major mechanism involves viral binding to the ACE2 receptor, expressed in pancreatic β-cells, hepatocytes, and adipose tissue [28]. Viral entry may exert direct cytotoxic effects, impairing insulin production and secretion. Systemic inflammation and the acute “cytokine storm” may further aggravate insulin resistance, leading to disruption of glucose metabolism [14,15].

Several studies have shown that SARS-CoV-2 infection can significantly disrupt glucose metabolism, leading to hyperglycemia, insulin resistance, and in some cases, new-onset diabetes, even in individuals without prior metabolic disorders. These alterations may persist beyond the acute phase, contributing to post-acute sequelae (PASC, or “long COVID”) [9,10,11,12,13]. SARS-CoV-2 may also stimulate glycolysis in monocytes, increasing lactate production and consequently serum LDH levels, which are characteristic of severe forms [25]. For example, Chen et al. (2022) demonstrated that even patients with mild disease exhibited persistent metabolic alterations 2–3 months after recovery (elevated fasting glucose and reduced insulin sensitivity) [23]. Similarly, Montefusco et al. described persistent hyperglycemia and altered insulin secretion dynamics during convalescence, suggesting that the virus may act both as a trigger and as an accelerator of metabolic dysfunction [29].

### 4.6. Treatments and Their Interaction with COVID-19-Related Hyperglycemia

Pharmacological treatments administered during the COVID-19 pandemic may significantly influence glucose homeostasis, either directly through metabolic effects or indirectly as a reflection of disease severity.

In our cohort, remdesivir was prescribed significantly more often in hyperglycemic patients (31.7% vs. 15.3%; *p* < 0.001), consistent with their more severe clinical presentation and greater need for oxygen therapy. This aligns with previous findings indicating that remdesivir is more frequently administered in patients with advanced disease, where hyperglycemic complications are also more common [69]. Conversely, lopinavir/ritonavir (Kaletra) was used more frequently in normoglycemics (43.3% vs. 28.3%; *p* < 0.001), largely reflecting the therapeutic context of the early pandemic phases, when hyperglycemia prevalence was lower and Kaletra was still widely available and recommended. Favipiravir was prescribed more often in hyperglycemics (35.1% vs. 27.3%), although this difference did not reach statistical significance. Its increased use in later pandemic waves for high-risk populations likely reflects both clinical need and resource limitations. Nevertheless, a meta-analysis confirmed that favipiravir did not significantly reduce ICU admissions or oxygen therapy requirements [46].

Immunomodulatory therapies were also disproportionately prescribed in hyperglycemics, with tocilizumab and anakinra being significantly more common in this group (16.5% and 26.2% vs. 6.5% and 12.4%; *p* < 0.001). These data indicate that hyperglycemia frequently coexisted with a more severe inflammatory phenotype, often requiring IL-6 or IL-1 blockade to control cytokine storm [70,71]. Prior studies demonstrated that hyperglycemia contributes to systemic inflammation, endothelial dysfunction, and immune dysregulation, thereby intensifying the inflammatory cascade of COVID-19 [27,30,72]. Clinical evidence suggests that such interventions may improve outcomes, with reduced oxygen requirements and shorter hospitalizations in selected patients [47].

Corticosteroid therapy, the standard of care for moderate-to-severe COVID-19, represents a double-edged sword in the context of glycemic control. While corticosteroids reduce mortality by attenuating inflammation, they are a well-recognized cause of hyperglycemia [72,73]. In our study, 40% of patients who were normoglycemic at admission developed hyperglycemia during hospitalization, and 94.5% of these cases were associated with corticosteroid therapy. Importantly, newly developed hyperglycemia was not consistently linked with poor prognosis (only 16.2% progressed to severe/critical forms, and 0.9% required ICU admission). However, persistent dysglycemia was observed in nearly one-third of patients at discharge (29.3% with glucose > 140 mg/dL), raising concern for long-term metabolic sequelae and highlighting the need for continued follow-up [71,72].

Beyond acute COVID-19 therapies, pharmacological treatments used for comorbid conditions may further influence glucose metabolism. Beta-blockers, antipsychotics, and certain chemotherapy agents are known to alter insulin sensitivity and impair glucose regulation [38,43]. Although detailed data on chronic treatments were not available in our dataset, the presence of comorbidities such as hypertension, obesity, or cancer in our sample suggests that their pharmacological management may have contributed to the observed dysglycemia. This represents a limitation of the present study but also underscores the importance of evaluating chronic medication profiles in future prospective research.

Taken together, our findings support the concept that admission hyperglycemia is not only a marker of COVID-19 severity but also a consequence of interactions between the viral pathophysiology, host comorbidities, and therapeutic interventions. The frequent use of antivirals and immunomodulators in hyperglycemics reflects disease severity, whereas corticosteroid therapy represents a direct iatrogenic driver of dysglycemia. Prospective studies are needed to clarify the extent to which acute COVID-19 therapies and long-term pharmacological treatments contribute to new-onset or persistent hyperglycemia, and whether stricter glycemic monitoring and targeted interventions could mitigate these risks.

### 4.7. Length of Hospital Stay and Costs

Patients with admission hyperglycemia had a mean hospital stay approximately two days longer than normoglycemics (12.14 vs. 10.1 days; *p* < 0.001), suggesting that hyperglycemia may serve as a marker of more severe disease or delayed recovery. The risk of prolonged hospitalization was significantly higher among hyperglycemics (HR = 1.72; *p* < 0.001). At the same time, the mean hospitalization costs were about EUR 800 higher per patient (EUR 1846 vs. 1043; *p* < 0.001), reflecting longer admissions, the need for additional investigations, more expensive therapies, and a higher probability of complications [38].

The mean time from symptom onset to admission did not differ significantly between groups (6.8 vs. 6.3 days; *p* = 0.087), suggesting that differences in hospital stay and costs cannot be attributed to delayed presentation but rather to disease severity and management complexity. Similarly, a study of 1122 patients across 88 U.S. hospitals reported that those with diabetes or uncontrolled hyperglycemia at admission had higher mean glucose levels (202 vs. 114 mg/dL; *p* < 0.001), increased mortality (28.8% vs. 6.2%; *p* < 0.001), and longer hospital stays (5.7 vs. 4.3 days), supporting the association of hyperglycemia with more severe disease and a more complex clinical course [73].

### 4.8. Limitations and Future Directions

This study has several limitations. First, the retrospective and observational design precludes establishing a causal relationship between admission hyperglycemia and COVID-19 outcomes and may introduce recording bias. Second, as a single-center study conducted during a specific period (August 2020–July 2021), the results may not be generalizable to other populations, regions, viral variants, or therapeutic regimens. In the absence of data on HbA1c, C-peptide, or pancreatic autoantibodies, it was not possible to differentiate the underlying mechanism of hyperglycemia (stress-induced vs. insulin resistance vs. autoimmune insulin deficiency). Likewise, a clear distinction between stress hyperglycemia and newly diagnosed diabetes could not be made.

Moreover, the potential impact of pharmacological treatments on glucose metabolism represents an additional limitation of our study. While the role of corticosteroids in inducing hyperglycemia is well established and was frequently observed in our cohort, the contribution of other chronic medications such as beta-blockers, antipsychotics, or chemotherapy agents could not be assessed due to lack of detailed data. This restricts our ability to fully disentangle the relative contributions of acute COVID-19-related dysglycemia versus iatrogenic or comorbidity-related factors. Future prospective studies should systematically record chronic medication use and its metabolic impact.

Nevertheless, the findings indicate admission hyperglycemia as a clinically relevant and easily measurable prognostic marker. Hyperglycemia at admission may reflect both an accentuated systemic inflammatory response and pre-existing metabolic imbalance, being associated with more severe forms of disease, prolonged clinical course, and higher costs [74]. New-onset hyperglycemia and diabetes increase cardiovascular risk and mortality if not managed promptly [16,17]. Systematic monitoring of glucose levels at admission and the implementation of careful glycemic control could enable early identification of high-risk patients, optimize therapeutic strategies, and improve resource allocation. Prospective studies and clinical trials are needed to determine whether early intervention and strict glycemic control can improve outcomes and reduce complications, thereby consolidating hyperglycemia as an integrated prognostic factor in COVID-19 management.

## 5. Conclusions

**Figure 10:**
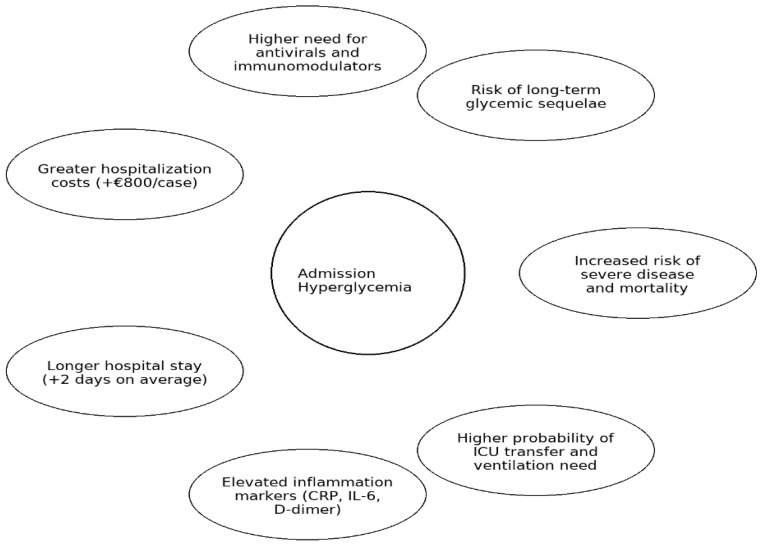
Graphical summary of the main clinical and economic implications of admission hyperglycemia in COVID-19. Legend: Graphical summary of the main clinical and prognostic implications of admission hyperglycemia in hospitalized COVID-19 patients, including disease severity, treatment needs, inflammatory profile, hospitalization outcomes, and long-term metabolic risks.

Admission hyperglycemia was associated with increased disease severity, respiratory complications, inflammatory dysregulation, prolonged hospital stay, higher costs, and worse prognosis, even in patients without pre-existing diabetes (Figure 10).

These findings indicate that admission hyperglycemia represents an early and easily measurable prognostic marker, supporting the need for systematic glucose monitoring and targeted metabolic management in COVID-19.

## Figures and Tables

**Table 1:** Epidemiological and clinical variables assessed and rationale for inclusion.

Variable	Category	Rationale
Age, sex	Demographic	Established determinants of COVID-19 severity [14,15]
Cardiovascular disease (hypertension, myocardial infarction with stent)	Medical history	Common comorbidities linked with worse outcomes [41,42]
Pulmonary disease (asthma, COPD)	Medical history	Associated with respiratory compromise [38]
Obesity, hepatic steatosis	Metabolic history	Known risk factors for severe COVID-19 [17,43]
Chronic kidney disease	Medical history	Associated with poor prognosis [43]
Clinical form of COVID-19, initial symptoms, time from symptom onset to admission	Clinical	Indicators of disease onset and progression [44]
Oxygen therapy, CPAP requirement, antiviral and immunomodulatory treatments (remdesivir, tocilizumab, anakinra), insulin therapy	Clinical management	Reflect severity and treatment intensity [45,46,47]
Hematological and biochemical parameters (leukocytes, lymphocytes, platelets, CRP, ferritin, ESR, fibrinogen, D-dimer, IL-6, AST, ALT, LDH, creatinine)	Laboratory	Reflect systemic inflammation, tissue injury, coagulation status, and organ dysfunction [26,48]
Chest radiography/CT findings	Imaging	Quantifies pulmonary involvement [49]
Length of stay, ICU transfer, hospitalization costs	Outcomes	Indicators of healthcare burden and prognosis [50]

**Table 2:** Demographic characteristics and comorbidities in patients with hyperglycemia versus normoglycemia at hospital admission.

Parameter	Hyperglycemia (*n* = 734)	Normoglycemia (*n =* 275)	*p*-Value
Sex			0.0003 *
Male (%)	428 (58.3%)	125 (45.5%)	
Female (%)	306 (41.7%)	150 (54.5%)	
Comorbidities			
Arterial hypertension	376 (51.2%)	114 (41.5%)	0.0070
Myocardial infarction/stent	21 (2.9%)	4 (1.5%)	0.2926
COPD	14 (1.9%)	4 (0.4%)	0.8283
Bronchial asthma	17 (2.3%)	9 (3.3%)	0.5281
Malignant neoplasms	8 (1.1%)	2 (0.7%)	0.8721
Obesity	79 (10.8%)	10 (3.6%)	0.0006
Hepatic steatosis	22 (3.0%)	5 (1.8%)	0.4154

Legend: Data are presented as numbers of patients (percentage). *p*-values were calculated using the Chi^2^ test. Abbreviations: COPD = chronic obstructive pulmonary disease. * *p*-value refers to overall sex distribution between groups.

**Table 3:** Age distribution by admission glycemic status.

Age Group (Years)	Hyperglycemia (*n* = 734)	Normoglycemia (*n* = 275)
18–35	20 (2.7%)	21 (7.6%)
36–50	129 (17.6%)	68 (24.7%)
51–65	307 (41.8%)	113 (41.1%)
66–80	278 (37.9%)	73 (26.5%)

Legend: Distribution of age groups among patients with admission hyperglycemia and normoglycemia. Data are expressed as number of patients (percentage of group total). No statistical test was applied, as values are descriptive.

**Table 4:** Comparison of symptom prevalence between hyperglycemic and normoglycemic COVID-19 patients.

Symptom	Hyperglycemia (*n* = 734)	Normoglycemia (*n* = 275)	*p*-Value
Fever	534 (52.9%)	196 (19.4%)	0.697453
Cough	491 (48.7%)	164 (16.3%)	0.037819
Dyspnea	347 (34.4%)	76 (7.5%)	<0.001
Fatigue	339 (33.6%)	118 (11.7%)	0.389855
Myalgia	191 (18.9%)	69 (6.8%)	0.825703
Headache	173 (17.1%)	81 (8.0%)	0.066295
Sore throat	16 (1.6%)	11 (1.1%)	0.168757
Anosmia/Ageusia	37 (3.7%)	23 (2.3%)	0.066097
GI symptoms	137 (13.6%)	66 (6.5%)	0.072793

Legend: Data are presented as numbers of patients (percentage). *p*-values were calculated using the Chi^2^ test. Abbreviations: GI = gastrointestinal symptoms (nausea, vomiting, diarrhea).

**Table 5:** Age-stratified CPAP requirement in COVID-19 patients according to glycemic status at admission.

Age Group (Years)	Hyperglycemic CPAP	Normoglycemic CPAP	*p*-Value
18–35	0/20 (0.0%)	0/21 (0.0%)	-
36–50	8/129 (6.2%)	1/68 (1.5%)	0.2488
51–65	24/307 (7.8%)	1/113 (0.9%)	0.0150
66–80	37/278 (13.3%)	2/73 (2.7%)	0.0188

Legend: Data are presented as numbers of patients requiring CPAP/total number in age group (percentage). *p*-values were calculated using the Chi^2^ test. Abbreviation: CPAP = continuous positive airway pressure.

**Table 6:** Comparison of clinical characteristics between hyperglycemic and normoglycemic patients.

Clinical Parameter	Hyperglycemia (*n* = 734)	Normoglycemia (*n* = 275)	*p*-Value
Acute respiratory failure	497 (67.7%)	105 (38.2%)	<0.001
CPAP therapy	69 (9.4%)	4 (1.5%)	<0.001
Mild clinical form	106 (14.4%)	111 (40.4%)	<0.001
Moderate clinical form	282 (38.4%)	94 (34.2%)	0.2151
Severe clinical form	295 (40.2%)	66 (24.0%)	<0.001
Critical clinical form	50 (6.8%)	3 (1.1%)	0.0003
Transfer to ICU	48 (6.5%)	4 (1.5%)	0.0011
Death	28 (3.8%)	3 (1.1%)	0.0256

Legend: Data are presented as numbers of patients (percentage). *p*-values were calculated using the Chi^2^ test. Abbreviations: CPAP = continuous positive airway pressure; ICU = intensive care unit.

**Table 7:** Comparison of hospital stay duration and hospitalization costs between patients with hyperglycemia and normoglycemia at admission.

Variable	Hyperglycemia (*n* = 734)	Normoglycemia (*n* = 275)	*p*-Value
Mean hospital stay duration	12.14 days	10.1 days	<0.001
Mean hospitalization cost per case	€1846	€1043	<0.001

Legend: Data are expressed as mean values for hospital stay duration (days) and mean hospitalization cost per case (€) in patients with hyperglycemia versus normoglycemia at admission.

**Table 8:** Laboratory abnormalities in patients with hyperglycemia versus normoglycemia at hospital admission.

Parameter	Hyperglycemia (*n* = 734)	Normoglycemia (*n* = 275)	*p*-Value (Chi^2^ Test)
Leukocytes < 4000/μL	146 (18.5%)	66 (19.8%)	0.619
Lymphocytes < 1000/μL	470 (59.6%)	77 (23.1%)	<0.001
Platelets < 150,000/μL	112 (14.2%)	46 (13.8%)	0.878
Eosinophils = 0/μL	664 (84.2%)	139 (41.7%)	<0.001
ESR > 10 mm/h	647 (82.1%)	224 (67.2%)	<0.001
CRP > 10 mg/L	604 (76.6%)	200 (60.6%)	<0.001
Ferritin > 250 ng/mL	644 (81.7%)	188 (56.4%)	<0.001
LDH > 245 U/L	705 (89.4%)	260 (78.1%)	<0.001
D-dimers > 243 ng/mL	494 (62.6%)	164 (49.2%)	<0.001
Fibrinogen > 450 mg/dL	465 (59.0%)	151 (45.3%)	<0.001
ALT (GPT) > 45 U/L	383 (48.6%)	101 (30.3%)	<0.001
AST (GOT) > 45 U/L	304 (38.5%)	85 (25.5%)	<0.001
Creatinine > 1.2 mg/dL	155 (19.6%)	55 (16.5%)	0.250

Legend: Data are presented as numbers of patients (percentage). *p*-values were calculated using the Chi^2^ test. Abbreviations: ESR = erythrocyte sedimentation rate; CRP = C-reactive protein; LDH = lactate dehydrogenase; ALT (GPT) = alanine aminotransferase; AST (GOT) = aspartate aminotransferase.

**Table 9:** Comparison of antiviral treatments used in COVID-19 between hyperglycemic and normoglycemic patients.

Antiviral Treatment	Hyperglycemia (*n* = 734)	Normoglycemia (*n* = 275)	*p*-Value
Remdesivir	233 (31.7%)	42 (15.3%)	<0.001
Kaletra	208 (28.3%)	119 (43.3%)	<0.001
Plaquenil	152 (20.7%)	71 (25.8%)	0.0976
Favipiravir	258 (35.1%)	75 (27.3%)	0.0217
No antiviral treatment	20 (2.7%)	23 (8.4%)	<0.001

Legend: Data are presented as numbers of patients (percentage). Statistical significance was assessed using the Chi^2^ test.

**Table 10:** Administration of immunomodulatory treatment depending on glycemic status upon admission.

Immunomodulator Treatment	Hyperglycemic (*n* = 734)	Normoglycemic (*n* = 275)	*p*-Value (χ^2^ Test)
Tocilizumab (IL-6 inhibitor)	121 (16.48%)	18 (6.54%)	<0.001
Kineret (IL-1 inhibitor)	192 (26.15%)	34 (12.36%)	<0.001

Legend: Data are presented as numbers of patients (percentage). Statistical significance was assessed using the Chi^2^ test.

**Table 11:** Steroid use and new-onset hyperglycemia among normoglycemic patients at admission.

Variable	Normoglycemics Who Developed Hyperglycemia (n, %)
Developed hyperglycemia during hospitalization	110 (40.0%)
Received corticosteroids	104 (94.5%)
Progressed to severe/critical disease	18 (16.2%)
Required non-invasive ventilation/ICU transfer	1 (0.9%)
Glucose > 140 mg/dL at discharge	29.3%

Legend: Data are presented as numbers of patients (percentage) among normoglycemic individuals at admission who developed hyperglycemia during hospitalization.

**Table 12:** Comparative analysis of Spearman correlations across glycemic groups.

No.	Domain/Observation	Blood Glucose ≥ 300 mg/dL	Blood Glucose 181–300 mg/dL	Blood Glucose 107–180 mg/dL	Significant Correlation (Spearman)
1	Symptomatology	Dyspnea-myalgia, fever-ferritin	Dry cough	Dry cough	Yes
2	Chest X-ray (CXR)	CXR with ferritin, CRP, D-dimer	CXR with saturation	CXR with LDH, saturation	Yes
3	Oxygen saturation	Sat. with CXR, LDH, CRP	Sat. with CXR, LDH	Sat. with CXR, LDH	Yes
4	Systemic inflammation (CRP, ESR, fibrinogen)	CRP with O_2_ sat., CXR, D-dimer	CRP-ESR, CRP-fibrinogen	CRP-ESR, CRP-fibrinogen	Yes
5	Inflammation/Ferritin	Ferritin with CXR, fever	Ferritin with AST, ALT, LDH	Ferritin with LDH, CRP, AST	Yes
6	CRP and LDH	Not observed	Not observed	Strong positive correlation	Yes
7	Ferritin and LDH	Not observed	Correlation present	Correlation present	Yes
8	D-dimer	D-dimer with symptom onset days, CXR	D-dimer with age	D-dimer with LDH	Yes
9	LDH (tissue injury)	LDH with O_2_ sat., dyspnea	LDH with AST	LDH with AST, CRP, CXR, ferritin	Yes
10	Liver involvement (AST/ALT)	Not observed	ALT-AST	ALT-AST, AST-ferritin, AST-LDH	Yes

Legend: Correlation patterns between clinical, radiological, and laboratory parameters in COVID-19 patients stratified by admission blood glucose levels. Data summarize the main associations observed for each glycemic subgroup (≥300 mg/dL, 181–300 mg/dL, and 107–180 mg/dL), highlighting links between symptomatology, imaging findings, oxygen saturation, and markers of systemic inflammation, tissue injury, and liver involvement. Correlations are based on Spearman’s rank test. “Yes” indicates statistical significance (*p* < 0.05).

## Data Availability

The datasets analyzed during the current study are available from the corresponding author on reasonable request.

## Abbreviations

ACE2	Angiotensin-Converting Enzyme 2
ALT	Alanine Aminotransferase
AST	Aspartate Aminotransferase
COPD	Chronic Obstructive Pulmonary Disease
COVID-19	Coronavirus Disease 2019
CPAP	Continuous Positive Airway Pressure
CRP	C-Reactive Protein
CT	Computed Tomography
DM	Diabetes Mellitus
ESR	Erythrocyte Sedimentation Rate
ICU	Intensive Care Unit
LDH	Lactate Dehydrogenase
SARS-CoV-2	Severe Acute Respiratory Syndrome Coronavirus 2
